# The Evolution of the Linkage Among Geopolitical Risk, the US Dollar Index, Crude Oil Prices, and Gold Prices at Multiple Scales: A Wavelet Transform-Based Dynamic Transfer Entropy Network Method

**DOI:** 10.3390/e27111177

**Published:** 2025-11-20

**Authors:** Hanru Yang, Sufang An, Zhiliang Dong, Xiaojuan Dong

**Affiliations:** 1 School of Urban Geology and Engineering, Hebei GEO University, Shijiazhuang 052160, China; 2 School of Management, Hebei GEO University, Shijiazhuang 052160, China; 3 Strategy and Management Base of Mineral Resources in Hebei Province, Hebei GEO University, Shijiazhuang 052161, China; 4 Hebei Key Laboratory of Geotechnical Engineering Safety and Deformation Control, Cangzhou 061001, China

**Keywords:** transfer entropy network, MODWT, geopolitical risk, US dollar index, oil time series, gold time series, Network Topology, statistics

## Abstract

In recent years, the correlation mechanisms between geopolitical risks and financial markets have drawn considerable attention from both academic circles and investment communities. However, their multiscale, nonlinear interactive characteristics still require further investigation. To address this, this paper proposes a dynamic nonlinear causal information network combined with a wavelet transform model and the transfer entropy method. We select the geopolitical risk index, the US dollar index, Brent and WTI crude oil prices, COMEX gold futures, and London gold prices time series as the research objects. The results suggest that the network’s structure changes with time at different time scales. On the one hand, COMEX gold (London gold) acts as the major causal information transmitter (receiver) at all scales; both of their highest values appear at the mid-scale. The US dollar index plays a bridging role in information transmission, and this mediating ability decreases with increasing time scales. On the other hand, the fastest speed of causal information transmission is at the short scale, and the slowest speed is at the mid-scale. The complexity and systematic risk of causal network decrease with increasing time scales. Importantly, at the short-scale (D1), the information transmission speed slowed during the Russian–Ukrainian conflict and further decreased after the start of the Israel–Hamas conflict. Systematic risk has increased annually since 2018. This study provides a multiscale perspective to study the nonlinear causal relationship between geopolitical risk and financial markets and serves as a reference for policy-makers and investors.

## 1. Introduction

The investigation of causal relationships between geopolitical risk and financial markets has become of increasing importance in recent years [1,2,3]. This heightened focus is largely due to the frequent occurrence of geopolitical events, such as the Russia–Ukraine conflict and the Israel–Hamas conflict [4,5,6]. Studies have shown that geopolitical events can significantly affect financial markets by affecting supply and demand behavior [7], investor sentiment [8], and so on. Crude oil, as a major global commodity, plays a crucial role in both production and consumption, especially as economic globalization deepens [9,10], and is an important raw material in global industrial production. Russia and Israel’s neighboring regions are significant players in the import and export of crude oil, while the US dollar is the main currency for crude oil trading, indicating that there is a close relationship among crude oil price, the US dollar, and geopolitical risk. Additionally, as an investment product, crude oil has a strong financial attribute. Gold, characterized by its properties as a precious metal and a form of currency, serves as a means of value preservation [11], and is recognized as an effective hedging tool against significant fluctuations in crude oil prices [12,13]. Crude oil or gold prices affect the US dollar exchange rate through channels such as trade and asset investment [14,15]. Geopolitical risk has been demonstrated to exert a significant influence on the volatility of key financial assets, including the US dollar exchange rate, crude oil prices, and gold prices. Due to the time-varying nature of geopolitical risk, the causal relationships between geopolitical risk and these financial markets may exhibit nonlinear and uncertain characteristics [16,17,18]. Additionally, the fluctuation of financial markets also depends on the investment behavior of diverse market participants. Specifically, policy-makers usually pay attention to the long-scale perspective and market stability, while investors are more concerned with short-scale price fluctuations to maximize profits. Consequently, it is necessary to provide new evidence for the dynamic nonlinear causal relationships among geopolitical risk, crude oil, the US dollar, and gold from different time scales.

Many studies have investigated the causal relationship between geopolitical risk and financial markets. Shahbaz et al. [19] used the Granger test to explore the causality between geopolitical risk and oil prices, which provide a reference for studying the influence of geopolitical risk on the oil market. Su et al. [20] adopted a rolling window and the Granger approach to investigate the causal relationship between geopolitical risk and crude oil, reflecting that the relationship is dynamic. Zhang et al. [21] studied the heterogeneity of time-varying causality between geopolitical risk and green financial markets, indicating that important causal relationships often occur during emergencies. Huang et al. [1] analyzed the causality between geopolitical risk and the gold market via the causality-in-quantiles method and proved that the influence of different kinds of political events on the gold market varies. Zhao et al. [22] used transfer entropy to analyze the dynamic causality and systematic risk of bulk commodity prices. Kara et al. [6] combined a rolling window and causal model to study the interactions among geopolitical risk, WTI, and gold prices. Duan et al. [23] evaluated the time-varying causal effects among geopolitical risk, crude oil, and the dollar rate. Qin et al. [24] discussed the Granger causality between Russia’s geopolitical situation and gold price. There is considerable research potential between geopolitical events and crude oil, gold, and the US dollar [1,6,19,23,24,25]. Above all, previous studies have focused on the linear or nonlinear causal relationships among multiple variables via econometric models or hybrid models based on complex networks from a signal time scale. Therefore, a new model needs to be proposed to study the dynamic nonlinear causal relationships among multiple variables at multiple time scales.

A hybrid model that combines a wavelet transform [26,27,28,29] with a complex network [30,31,32,33] is an effective tool studying the dynamic relationships among different variables at different time scales. For example, Sun et al. [34] used MODWT to reveal the nonlinear causality of the energy market at different time scales. Xi et al. [29] constructed a network of transmissions of crude oil prices to energy inventories on the basis of wavelet decomposition, and indicated that the impact of crude oil price fluctuations on energy stocks is heterogeneous, with the greatest long-scale impact. Reference [35] used MODWT and the complex network method to measure the dynamic spillover relationships among various crude oils. Wen et al. [36] used the MODWT-Vine quantile regression model to inform investors about the risk transfer path among the oil market, commodity market, and Chinese stock market from different investment time scales. Shao et al. [37] combined MODWT and Granger causality to study the influence of geopolitical risk on mineral prices at different scales. In summary, the wavelet transform is a good methodology for determining the linkages between cross-markets with multiple time scales [38,39,40,41]. Therefore, we combine the MODWT and transfer entropy network models to explore the change rule of causality with time at multiple scales.

This paper proposes a dynamic nonlinear causal information network that is combined with a wavelet transform model, the transfer entropy method, and complex network theory. Geopolitical risk, the US dollar index, and the price of crude oil and gold can be selected as sample data, and dynamic networks can be reconstructed to reveal the strength and direction of causal information transmission among variables at short, medium, and long scales and analyze their evolutionary process. The contribution of this paper is to combine the wavelet transform and time-varying transfer entropy model to construct a causal information network for geopolitical risk, the dollar index, and crude oil and gold prices. Then, dynamic causal information networks are constructed according to the rolling window algorithm and complex network theory. By identifying the importance of a single node and analyzing the topological characteristics of the whole network, the nonlinear causality among geopolitical risk, the dollar index, and crude oil and gold prices can be further studied.

The remainder of this article is organized as follows. Section 2 focuses on the relevant data and methods, including the data sources, the wavelet transform, and construction of a dynamic causal information network. Section 3 presents the analysis results. Finally, we summarize the conclusions and propose some suggestions.

## 2. Materials and Methods

### 2.1. Data

Following Caldara and Iacoviello’s method [42], this paper describes the international geopolitical risk situation through the geopolitical risk index (GPR). The world’s largest and most influential crude oil futures contracts are Brent and WTI. Their high liquidity and price transparency can well represent fluctuations in international crude oil prices. The gold futures contract with the largest trading volume is the New York Metal Exchange, and the London gold price is the world’s largest gold spot trading market. Therefore, the COMEX gold futures and London gold spot prices are selected to represent international gold futures and spot prices.

We select the GPR, the US Dollar Index, and the daily prices of Brent crude oil futures, WTI crude oil futures, COMEX gold futures, and London gold spot as sample data. The period ranges from 2 January 2014 to 15 July 2024, which covers the China–US trade war, the COVID-19 pandemic, and the Russian–Ukrainian conflict. The total number is 2505, which aligns dates and ignores missing data, including holidays. The GPR data are downloaded from the website “https://www.matteoiacoviello.com/gpr.htm (accessed on 20 July 2024)”. The remaining data are from the WIND database.

**Figure 1:**
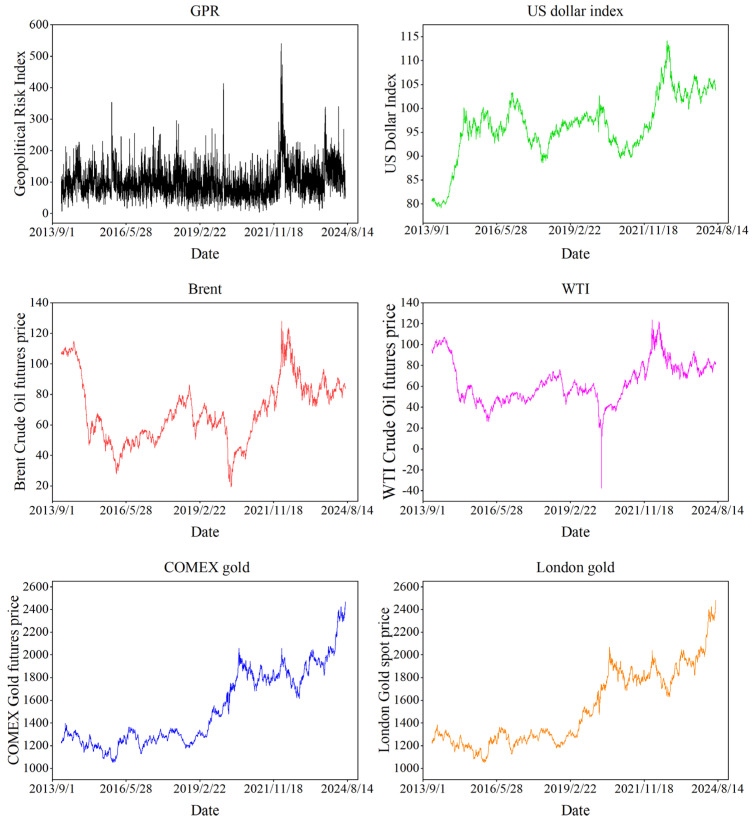
The sample data.

The fluctuation of each time series is shown in Figure 1, which indicates that the time period of all the variables is larger than 10 years. The distribution characteristics of these time series are shown in Table 1, which reveals that COMEX gold has the largest mean and the highest standard deviation. The US dollar index has a negative skewness value, whereas the other time series have positive skewness values. The US dollar index has the lowest absolute value of skewness, while GPR has the highest value. Moreover, the J-B test confirms the non-normal distribution of prices.

### 2.2. Methods

**Figure 2:**
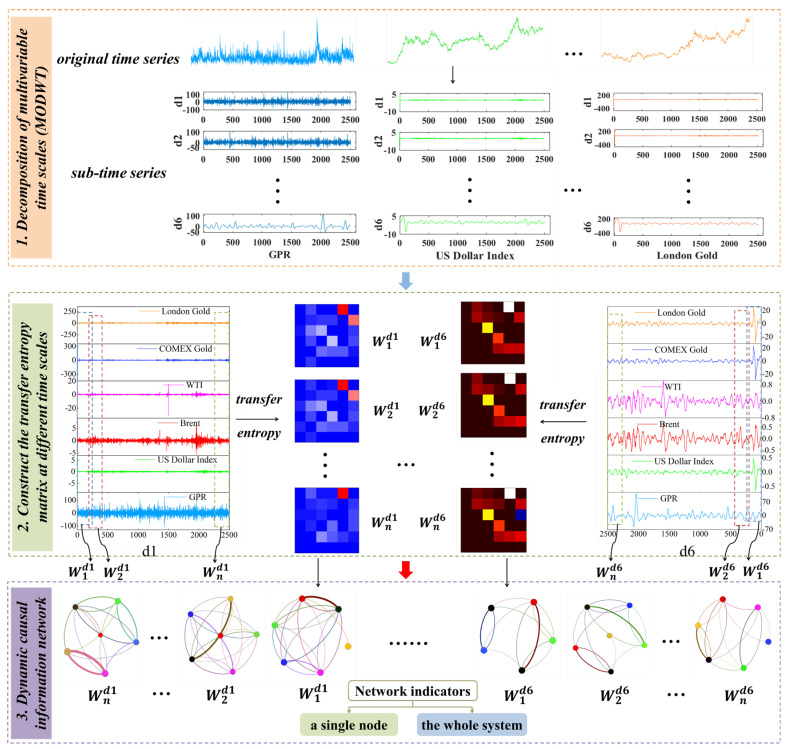
The model building process. Note: $W_{n}^{d_{m}}$ represents the n-th rolling window at the m-th time scale, where n = 2266, m = 1, 2, …, 6.

In order to reveal the dynamic evolution law and system dynamics characteristics of the nonlinear and multiscale causal relationship among geopolitical risk, US dollar index, and crude oil and gold prices. This paper proposes a causal information network based on the maximal overlap discrete wavelet transform model, transfer entropy model, and complex network theory to study the nonlinear causal relationships among GPR, the US dollar index, and the prices of crude oil and gold. First, the maximum overlap discrete wavelet transform model is used to decompose the original time series into sub-time series at multiple scales, aiming to explore the short-, medium- and long-scale evolutionary characteristics among variables. Second, the sub-time series on the same time scale can be mapped into a sequence of windows evolving into one another. Then, a transfer entropy matrix in a window can be obtained via the transfer entropy model, which represents the causal information transmission among variables. This suggests there is a sequence of transfer entropy matrices to describe the dynamic process of causal information transmission among these variables. Third, a transfer entropy matrix can be transferred into a directed weighted network referred to as a causal information network. The node in a network represents a sub-time series in a window, and the directed weighted edge is the causal information transmission from one sub-time series to another. The dynamic topology structure of a causal information network is investigated to identify important variables in the dynamic process at each time scale and to analyze the evolutionary structure of the network as a whole. The process is illustrated in Figure 2.

#### 2.2.1. Decomposition of Multivariable Time Scales (MODWT)

The wavelet transform is an effective method for analyzing unstable data and multiscale time series. It combines time and frequency information and positively affects non-stationary sequence analysis [43,44]. It can identify the volatility of time series [45]. Financial time series are essentially non-stationary, often exhibiting seasonal variations as well as long-term and short-term fluctuations. Many scholars have studied the relationships among variables by combining economic models and wavelet models [36,37]. We use the MODWT model [36] to decompose the original time series into sub-time series. The wavelet filter is composed of ${\tilde{h}}_{j,l}=\frac{h_{j,l}}{2^{j/2}}$, ${\tilde{g}}_{j,l}=\frac{g_{j,l}}{2^{j/2}}$, l(l=1,2…L) is the filter length, and j(j=1,2…J) is the number of scales. The wavelet coefficient ${\tilde{W}}_{j,l}$ and scale coefficient ${\tilde{V}}_{j,l}$ can be expressed as follows:

(1) $${\tilde{W}}_{j,l}=\frac{1}{2^{\frac{j}{2}}}\sum_{l=0}^{L-1}{\tilde{h}}_{j,l}X\left(t-1\right)\;\;$$

(2) $${\tilde{V}}_{j,l}=\frac{1}{2^{\frac{j}{2}}}\sum_{l=0}^{L-1}{\tilde{g}}_{j,l}X\left(t-1\right)\;$$

The wavelet coefficients at each scale are the same length as the original time series *X*. The wavelet coefficient and scale coefficient are as follows:

(3) $${\tilde{W}}_{j}={\tilde{w}}_{j}X\;\;$$

(4) $${\tilde{V}}_{j}={\tilde{v}}_{j}X\;\;$$

The original time series are decomposed into multiple scales and a trend. The formula is as follows:

(5) $$X=\sum_{j=1}^{J}{{\tilde{w}}_{j}}^{T}{\tilde{W}}_{j}+{{\tilde{v}}_{j}}^{T}{\tilde{V}}_{j}=\sum_{j=1}^{J}{\tilde{D}}_{j}+{\tilde{S}}_{J}$$

where ${\tilde{D}}_{j}={{\tilde{w}}_{j}}^{T}{\tilde{W}}_{j}$ represents the details of the MODWT on the decomposition scale, ${\tilde{S}}_{J}$ is the trend level, and *J* is set to 6.

#### 2.2.2. Constructing a Sequence of Transfer Entropy Matrices at Different Time Scales

Previous models lack in-depth research on the nonlinear causal dynamics between variables. Therefore, we introduce the transfer entropy method to improve the study of causality. On the basis of information theory, Schreiber proposed the concept of transfer entropy (TE) [46] and judged the causal relationship between variables by detecting the change in information transfer according to the Markov property. This quantitatively represents the degree of confusion of the system, the amount of information contained in the random variable, and the degree of uncertainty. The more confused the system is, the greater the entropy value, and the greater the uncertainty of the random variables. As a nonlinear extension of Granger causality, TE naturally combines the linear and nonlinear information flows of time series [47,48], which is more effective at expressing the nonlinear characteristics of causal relationships among variables [49,50]. In addition to the application in time series, the entropy method is also involved in the field of artificial intelligence and goodness of fit test. For instance, in the field of integrated method, the entropy is employed to acquire the objective criteria weights [51] and individual objective attribute weights [52]. Furthermore, entropy serves as an effective tool in goodness of fit tests [53,54,55].

Given a discrete random variable *X* with probability distribution $p\left(x\right)$, the average number of bits required to optimally encode independent draws can be calculated as

(6) $$H(X)=-\sum_{x}p\left(x\right){log}_{2}p(x)$$

Shannon’s formula measures uncertainty, which increases with the number of bits needed to optimally encode a sequence of realizations of X. In order to measure the information flow between two time series, let X and Y denote two discrete random variables with marginal probability distributions $p\left(x\right)$ and $p\left(y\right)$, and joint probability $p\left(x,y\right)$, whose dynamical structures correspond to a stationary Markov process of order k (process X) and  l (process Y). The Markov property implies that the probability to observe *X* at time  t+1 in state x conditional on the k previous observations is

(7) $$p\left(x_{t+1}|x_{t},\ldots ,x_{t-k+1}\right)=p\left(x_{t+1}|x_{t},\ldots ,x_{t-k}\right)$$

The average number of bits needed to encode the observation in  t+1, once the previous k values are known, is given by

(8) $$h_{X}\left(k\right)=-\sum_{x}x_{t+1},x_{t}^{\left(k\right)}log(x_{t+1}|x_{t}^{\left(k\right)})$$

where $x_{t}^{\left(k\right)}=(x_{t},\cdots ,\;x_{t-k+1})$, $y_{t}^{\left(l\right)}=(y_{t},\cdots ,\;y_{t-l+1})$. In the bivariate case, information flow from process  Y to process  X is measured by quantifying the deviation from the generalized Markov property, which is denoted as

(9) $$p\left(x_{t+1}|x_{t}^{\left(k\right)}\right)=p\left(x_{t+1}|x_{t}^{\left(k\right)},y_{t}^{\left(l\right)}\right)$$

Relying on the Kullback–Leibler distance. The formula for Shannon transfer entropy is given by

(10) $${TE}_{Y\to X}=\sum p(x_{t+1\;},x_{t}^{\left(k\right)},y_{t}^{\left(l\right)})log\frac{p(x_{t+1\;}|x_{t}^{\left(k\right)},y_{t}^{\left(l\right)})}{p(x_{t+1\;}|x_{t}^{\left(k\right)})}$$

where ${TE}_{Y\to X}$ measures the information flow from Y to X. ${TE}_{X\to Y}$, as a measure for the information flow from X to Y, can be derived analogously. $p(x_{t+1\;}|x_{t}^{\left(k\right)},y_{t}^{\left(l\right)})$ is a joint conditional probability, which predicts the probability distribution of the future state $x_{t+1\;}$ of X when both the history $x_{t}^{\left(k\right)}$ of X and the history $y_{t}^{\left(l\right)}$ of Y are known. $p(x_{t+1\;}|x_{t}^{\left(k\right)})$ is a conditional probability, which predicts the probability distribution of the future state $x_{t+1\;}$ of X when only the history $x_{t}^{\left(k\right)}$ of X itself history is known. $p(x_{t+1\;},x_{t}^{\left(k\right)},y_{t}^{\left(l\right)})$ is the joint probability, which refers to the probability that three variables appear at the same time, of the weight factor. ∑ denotes the summation of all state combinations. The dominant direction of the information flow can be inferred by calculating the difference between ${TE}_{Y\to X}$ and ${TE}_{X\to Y}$.

Combined with the rolling window algorithm, the transfer entropy between variables under a series of windows is calculated, and at the 5% level, the effective transfer entropy (*Eff. TE*) with significant causal relationship is selected to form a matrix, as shown in Equation (11).

(11) $$Eff.TE=\left[\begin{array}{l} \begin{array}{c} t_{1,1}\\ t_{2,1}\\ t_{3,1} \end{array}\begin{array}{c} t_{1,2}\\ t_{2,2}\\ t_{3,2} \end{array}\begin{array}{c} t_{1,3}\\ t_{2,3}\\ t_{3,3} \end{array}\begin{array}{c} t_{1,4}\\ t_{2,4}\\ t_{3,4} \end{array}\begin{array}{c} t_{1,5}\\ t_{2,5}\\ t_{3,5} \end{array}\begin{array}{c} t_{1,6}\\ t_{2,6}\\ t_{3,6} \end{array}\\ \begin{array}{c} t_{4,1}\\ t_{5,1}\\ t_{6,1} \end{array}\begin{array}{c} t_{4,2}\\ t_{5,2}\\ t_{6,2} \end{array}\begin{array}{c} t_{4,3}\\ t_{5,3}\\ t_{6,3} \end{array}\begin{array}{c} t_{4,4}\\ t_{5,4}\\ t_{6,4} \end{array}\begin{array}{c} t_{4,5}\\ t_{5,5}\\ t_{6,5} \end{array}\begin{array}{c} t_{4,6}\\ t_{5,6}\\ t_{6,6} \end{array} \end{array}\right]$$

#### 2.2.3. Constructing a Dynamic Causal Information Network

**Figure 3:**
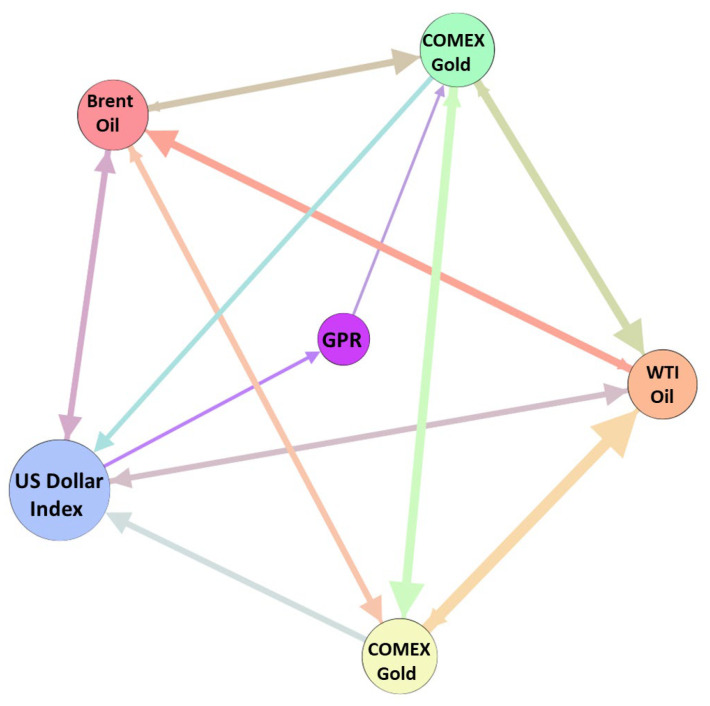
An example of causal information network. Notes: The node is the variable, the edge is the information transfer, and the color indicates that the source node; the larger the weighted out degree, the larger the node and the thicker the edge.

According to the transfer entropy matrix, a directed weighted complex network in each window is constructed. In the network, nodes are represented by variables. The effective transfer entropy value $t_{\left(x,y\right)}$ of node *X* and node *Y* is the weight of the edge, when *X* = *Y*, $t_{\left(x,y\right)}=0$. Figure 3 shows an example of a causal information network under a window in D1.

### 2.3. Measures of Causal Relationships

#### 2.3.1. Measure of a Single Node

In the network, the degree represents the number of edges directly connected to the node and other nodes, including the in degree and out degrees. The in degree is the number of edges that other nodes point to one node, while the out degree denotes the number of connected edges from one node to adjacent nodes. Therefore, in the dynamic causal information network, the degree’s direction represents the causality flow. The larger the degree of the node, the more edges of causal relationships there are between one variable and other variables. The calculation method is as follows:

(12) $$k_{i}^{in}=\sum_{j=1}^{N}a_{ji}$$

(13) $$k_{i}^{out}=\sum_{j=1}^{N}a_{ij}$$

The strength of the causal relationship between the variables can be measured by the strength; variables with higher strength values have a stronger causal relationship with other variables. The strength of a node represents the total weight of its edges. Strength is divided into in strength and out strength in a directed network. In this article, the in strength represents the causal information obtained by a variable from the other variables, and the out strength represents the intensity of the causal information influence of a variable on its neighbor variables. The calculation is as follows:

(14) $$S_{i}^{in}=\sum_{j=1}^{N}a_{ji}*w_{ji}$$

(15) $$S_{i}^{out}=\sum_{j=1}^{N}a_{ij}*w_{ij}$$

Betweenness centrality is the role of nodes as a bridge for information evolution between other nodes and reflects the participation of nodes in evolution and the ability to control information. The calculation is as follows:

(16) $${BC}_{i}\;=\;\frac{\sum_{j}^{n}\sum_{k}^{n}g_{jk}(i)/g_{jk}}{n^{2}-3n+2},\;j\neq k\neq i,j\;<k.$$

where $g_{jk}(i)$ is the frequency of occurrence in the shortest paths between nodes *j* and *k* passing through node  i, and $g_{jk}$ is the number of shortest paths connecting nodes j and k.

#### 2.3.2. Measuring the Dynamic Structure of a Causal Information Network as a Whole

The average path length (*L*) is an important index for evaluating information efficiency in a network. In the network, a node can reach another node through one or more different paths. The path with the least number of network edges between node i and node  j is called the shortest path ($d_{ij}$), and the average path length (*L*) is the average value of the shortest path between all nodes in the network. The maximum value of the shortest path ($d_{ij}$) in the network is the diameter of the network (**d**). The smaller the two indicators are, the better the integrity of the network, and the faster the causal relationship transmission between markets. The calculation formulas for the *L* and *D* are as follows:

(17) $$L=\frac{1}{n(n-1)}\sum_{i}\sum_{j}d_{ij}$$

(18) $$D=max(d_{ij})$$

We use the system risk entropy indicator to measure and characterize the systematic risk. This index is obtained mainly from the eigenvalues of the correlative matrix, which can explain the risk level of the whole system. The smaller the value of the system risk entropy, the higher the systematic risk. The specific definitions are as follows:

(19) $${RE}_{H}=(-\frac{1}{\log\left(N\right)})\times \sum_{i=1}^{H}\left(\frac{\lambda_{i}}{N}\right)\times log(\frac{\lambda_{i}}{N})$$

where $\lambda_{i}$ represents the eigenvalues that can indicate real market information, H represents the number of eigenvalues, and N represents the number of variables, ${0\leq RE}_{H}\leq 1$.

## 3. Results

Notably, in multi-resolution theory, the similarity between the decomposed and the original sequence increases as the time scale increases. The signal sequence fluctuates most violently at the D1 scale. As the time scale increases, the fluctuation becomes increasingly gentle, which indicates that at the D6 time scale, the original time series and the sub-time series have the greatest similarity. To ensure that all the components of the generated transfer entropy matrix are stationary, this paper uses the ADF test at the 5% significance level. According to the test results, all sub-time series from D1 to D5 satisfy the stability requirements, and the sub-series of other variables except the GPR subsequences at the D6 scale are stable after the first-order difference, which meets the requirements and, thus, can be used. According to the results of the kurtosis, skewness, and JB tests, all the time series data after MODWT decomposition have ‘peak and thick tail’ characteristics.

On the basis of the method discussed in Section 2, first, MODWT is used to reconstruct the series of geopolitical risk, dollar index, Brent crude oil price, WTI crude oil price, COMEX gold price, and London gold price into multiple sub-time series; since there are investors with different investment horizons in the market, they focus on different information scales. The number of time scale decomposition is set to 6 [43]. Because the time interval is days, the time period corresponding to the first layer scale is 2–4 days, the second layer scale is 4–8 days, and the third layer scale is 8–16 days. The fourth scale corresponds to 16–32 days, the fifth scale corresponds to 32–64 days, and the sixth scale corresponds to 64–128 days. D1, D2, D3, D4, D5, and D6 are the details of each layer, and S is the approximate value. The details are shown in Table 2. Second, the entire sequence of the same scale is divided into a sequence of fragments using a rolling window. The width of the window θ is 240 days, excluding holidays and rest days, equivalent to a one-year economic cycle; each scale has 2505-θ + 1 rolling windows. Following this, the transfer entropy matrix of geopolitical risk, the dollar index, and crude oil and gold prices under the action of the rolling window is subsequently calculated. Third, the causal information transmission matrix is transformed into a directed weighted network, the transmission networks of six sets of data at six time scales are obtained, and a sequence of networks is generated at each scale. By measuring the network indicators, the time-varying nonlinear causal relationships among geopolitical risk, the dollar index, and crude oil and gold prices are compared and analyzed from short-, medium- and long-scale perspectives.

### 3.1. The Number of Neighbors of the Risk Transmission of Different Variables

**Figure 4:**
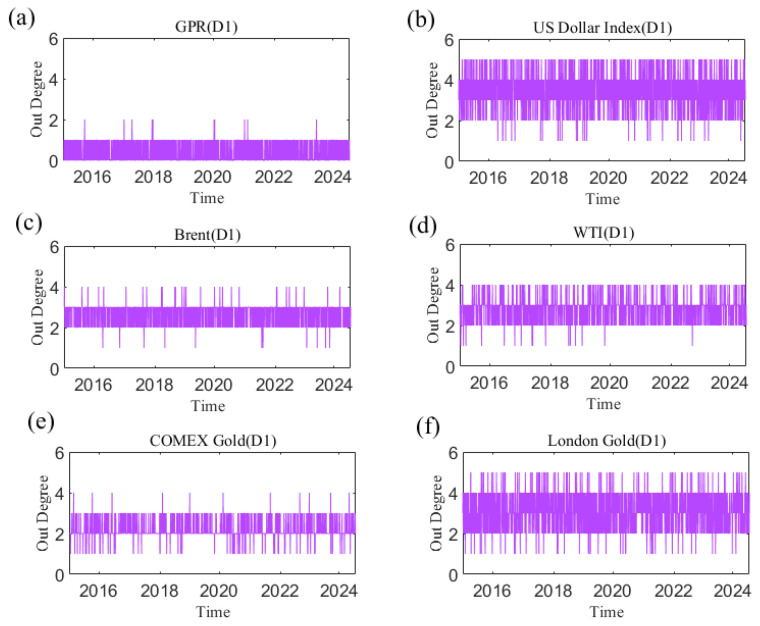
The out degree of each variable at the D1 scale varies with time. (**a**) geopolitical risk; (**b**) US Dollar Index; (**c**) Brent crude oil; (**d**) WTI crude oil; (**e**) COMEX gold; (**f**) London gold.

**Figure 5:**
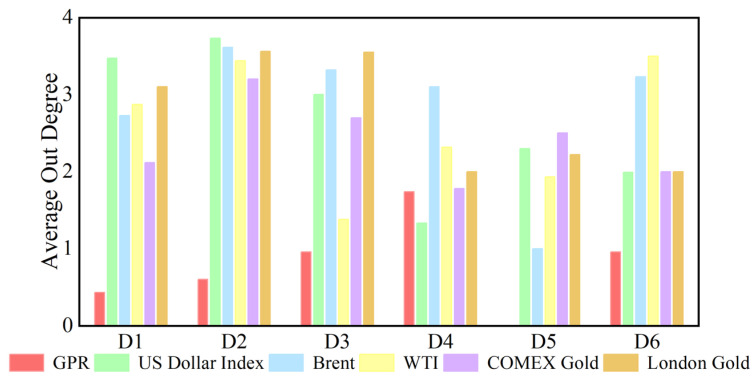
Average out degree of each series at different time scales.

The out degree for each sub-time series in causal information networks at the D1 time scale is shown in Figure 4. Appendix A presents the dynamic out degree of each variable at other time scales. The number of neighbors of a sub-time series as a causal information transmitter is time-varying at each time scale. The time-varying characteristics at different time scales of out degree are different, indicating that they are also related to time scales. Additionally, Figure 5 shows the average out degree of each variable at different time scales. For example, at the scale D1, the average out degree of the US dollar is the highest, and the average out degree of geopolitical risk is the smallest. This reflects that the US dollar has the largest number of neighbors as a causal information transmitter, which is most likely to affect other variables in the network, whereas the number of neighbors with geopolitical risk is the smallest. With increasing time scale, the average out-degree of the US dollar first decreases and then increases, and geopolitical risk generally shows an upward trend. This suggests that the number of neighbors of the US dollar as an information transmitter first increases but then decreases.

**Figure 6:**
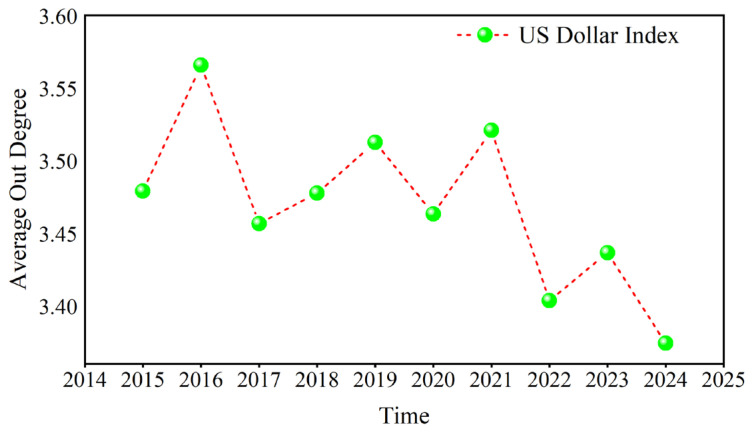
The annual average of the out degree.

Taking the US dollar index in D1 as an example, the average value of its annual out degree is calculated, as shown in Figure 6. The results indicate that the maximum value occurs in 2016, while the minimum occurs in 2024. Owing to the outbreak of the COVID-19 pandemic in 2020, global trading and investment activity decreased, which curbed the role of the US dollar, and then the US dollar’s out degree rose as the economy recovered. The out degree of the US dollar rapidly declined when the Russian–Ukrainian conflict occurred in 2022, reaching its lowest value in 2024.

**Figure 7:**
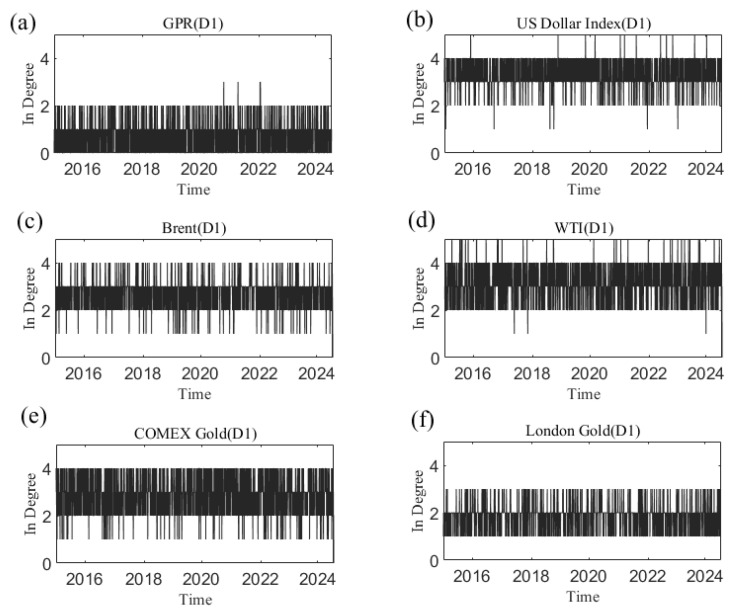
The in degree of each variable at the D1 scale varies with time. (**a**) geopolitical risk; (**b**) US Dollar Index; (**c**) Brent crude oil; (**d**) WTI crude oil; (**e**) COMEX gold; (**f**) London gold.

**Figure 8:**
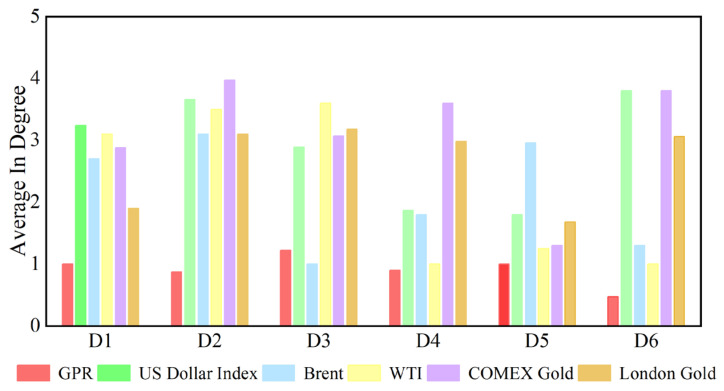
Average in degree of each series at different time scales.

Figure 7 shows the dynamic in degree for each variable at the D1 time scale. Appendix B shows the change in each variable’s in degree with time at other time scales. We can see that the number of causal information receivers changes over time on a time scale. The time-varying characteristics at different time scales differ in terms of degree. Additionally, the average degree of each variable at different time scales is reflected in Figure 8. For example, at scale D1, the average in degree of the US dollar is the greatest, and the average out degree of geopolitical risk is the smallest. The average in degree of geopolitical risk is the most stable at the D1–D6 scales, indicating that as the time scale changes, the number of neighbors of geopolitical risk as the receiver of causal information does not change significantly with increasing time scales.

**Figure 9:**
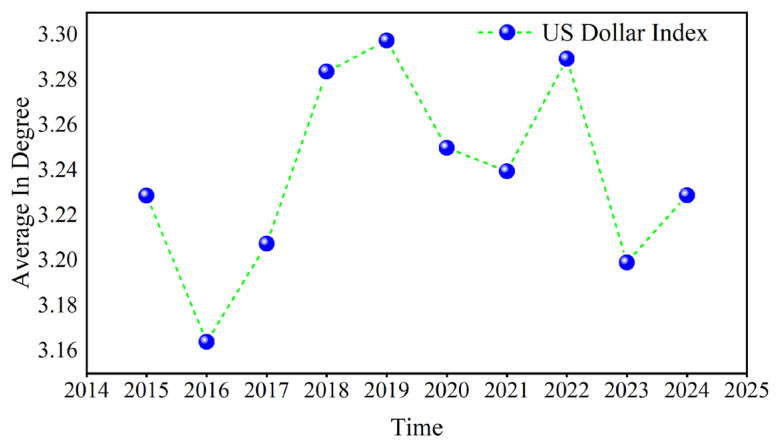
The annual average of the in degree.

Figure 9 takes the US dollar index in D1 as an example and calculates the average value of its annual in degree. The minimum value occurred in 2016, and the maximum value occurred in 2019. In 2015, the in degree of the US dollar reached its lowest point after the Chinese stock disasters and then continued to rise until the outbreak of the COVID-19 pandemic declined in 2020. The Russian–Ukrainian conflict in 2022 and the Israel–Hamas conflict in 2023 caused the in degree to increase again.

### 3.2. The Influence of Different Variables in Causal Information Network

**Figure 10:**
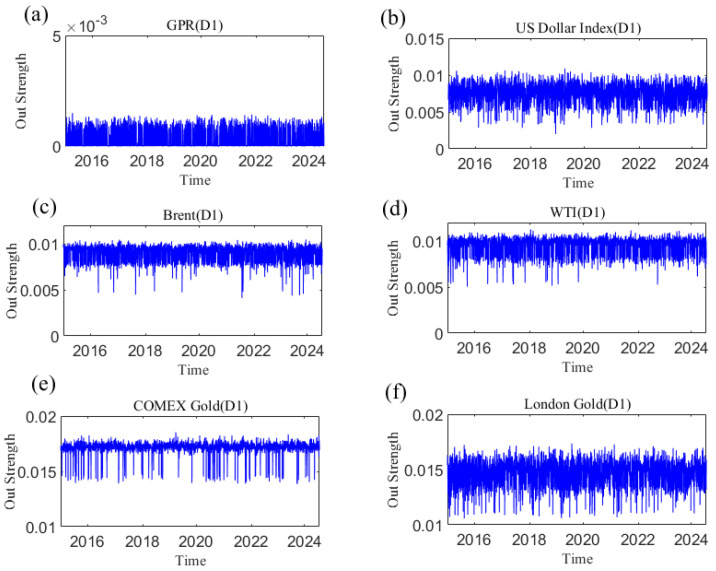
The out strength of each variable at the D1 scale varies with time. (**a**) geopolitical risk; (**b**) US Dollar Index; (**c**) Brent crude oil; (**d**) WTI crude oil; (**e**) COMEX gold; (**f**) London gold.

**Figure 11:**
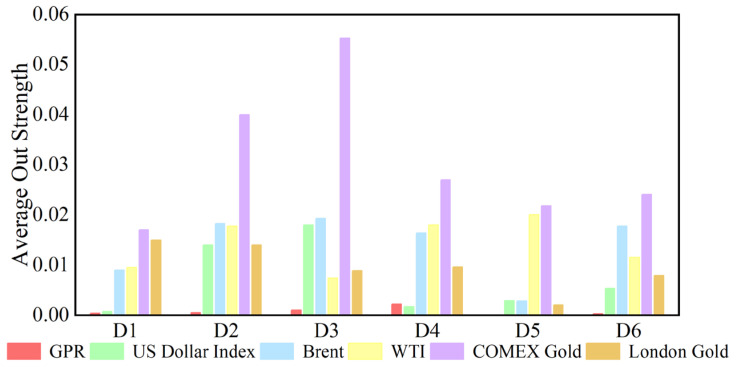
Average out strength of each series at different time scales.

In the analysis of this paper, the out strength measures the strength of a variable’s influence on other variables. The dynamic causal out strength of each variable at the D1 scale is shown in Figure 10, and the dynamic out strength of each variable at other time scales is shown in Appendix C. The out strength of causal information transmitted to others by geopolitical risks, the dollar index, and crude oil and gold prices all change over time. The time-varying characteristics of causal information outflow are different at different time scales, showing that its changes are related to time scales. Figure 11 shows the average out strength of the six variables at the D1–D6 scales. For example, COMEX gold has the largest value at the D1 scale and has the greatest influence on other variables in the past ten years. The out strength of geopolitical risk is the smallest at scale D1. The results show that gold is the most important causal information transmitter.

**Figure 12:**
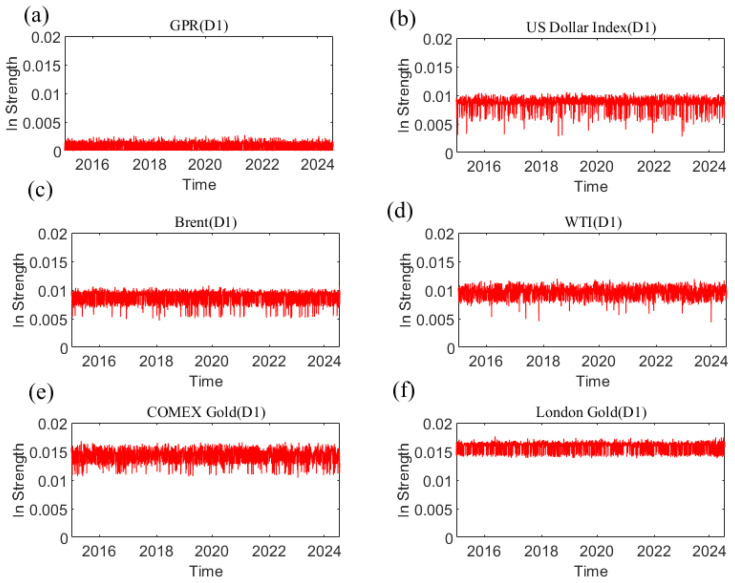
The in strength of each variable at the D1 scale varies with time. (**a**) geopolitical risk; (**b**) US Dollar Index; (**c**) Brent crude oil; (**d**) WTI crude oil; (**e**) COMEX gold; (**f**) London gold.

**Figure 13:**
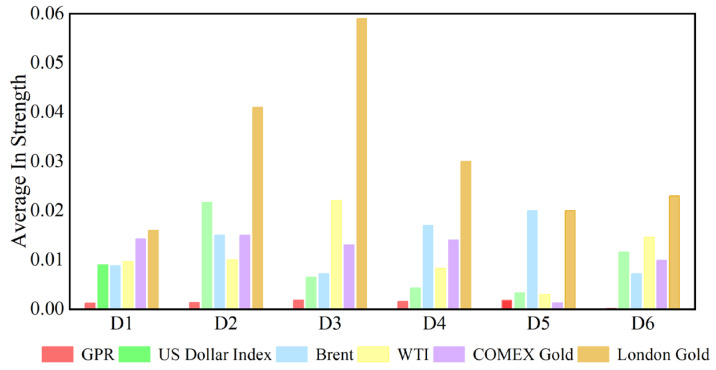
Average in strength of each series at different time scales.

The in strength measures how strongly each variable is affected by other variables. The greater the weight is, the easier it is to be influenced by other variables. Figure 12 shows the dynamic in strength of each variable at the D1 scale. Please refer to Appendix D to understand the dynamic in strength of each variable at other time scales. The results indicate that the in strength of geopolitical risk, the US dollar, and the price of crude oil and gold receiving causal information from others is time-varying. The dynamic characteristics of the in strength at different time scales are different, reflecting that the causal information inflow characteristics are related to the time scale. Figure 13 indicates the average in strength of the six variables at different scales. At scale D1, the average strength of London gold is the highest, indicating that London gold, as the receiver of dynamic causal information of other variables, has the greatest intensity and is most easily influenced by other variables, whereas the strength of geopolitical risk is the smallest. The combination of the out strength and in strength of each variable at the other scales in the Appendix suggests that the interaction among variables in the system gradually weakens as the time scale increases.

**Figure 14:**
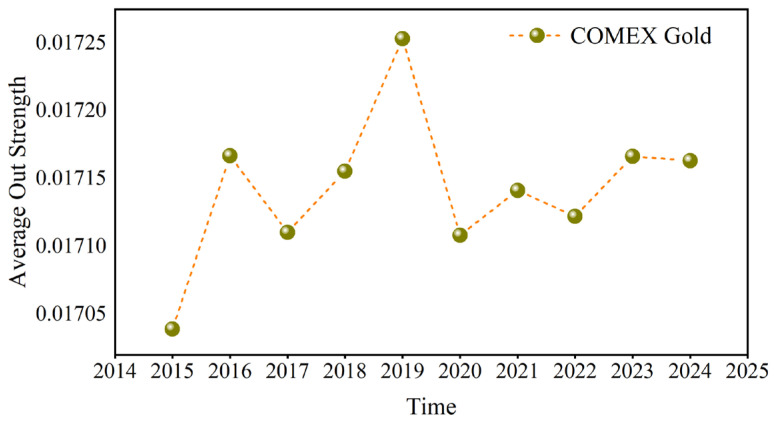
The annual average of the out strength.

**Figure 15:**
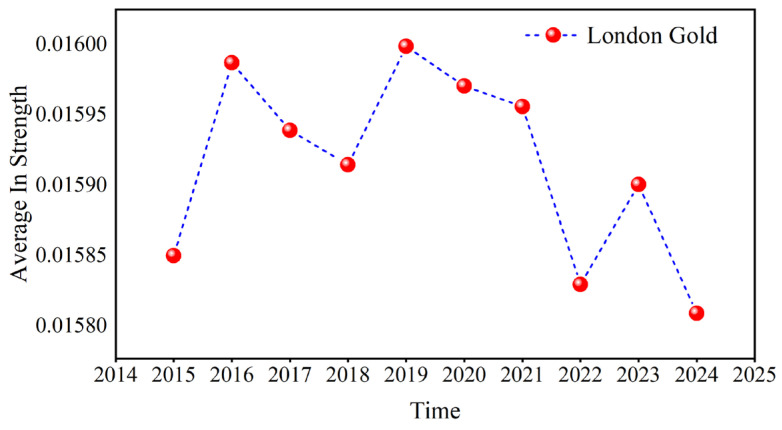
The annual average of the in strength.

Because COMEX gold has the largest out strength and London gold has the largest in strength, Figure 14 and Figure 15 use COMEX and London gold at the D1 scale, respectively, as examples to calculate their annual average values. Both their fluctuation rules are similar in the past ten years.

Russia’s annexation of Crimea in early 2014 triggered the crisis in Ukraine. This led to Western sanctions against Russia, which raised geopolitical risks. Concurrently, from 2014 to 2015, the Syrian civil war continued to intensify, attracting the participation of many countries and regions, such as Russia, the United States, and Saudi Arabia. The Middle East continues to face geopolitical tensions because of multiple conflicts, such as the ISIS threat in Iraq and the negotiation of the Iran nuclear deal. Market participants typically react to events such as geopolitical tensions, sanctions, and supply disruptions, resulting in volatility in crude oil prices, thereby enhancing the hedging effect of gold on the crude oil market and highlighting the dominance of gold. This is reflected in the sharp increase in the out strength and in strength of gold during 2015.

After the upward trend, some countries impacted by supply disruptions gradually resumed crude oil production, resulting in increased supply and thus lower crude oil prices. In addition, Donald Trump’s election as US President in late 2016 and early 2017 triggered significant changes and uncertainties in US domestic and international policies. For instance, the Trump administration adopted more protectionist trade policies, including the imposition of tariffs on Chinese goods. This might have affected world trade and the economy, which might then have affected crude oil demand. In 2018, Iran’s crude oil exports significantly decreased because of the United States’s reimposition of sanctions against the country. Crude oil production has decreased due to Venezuela’s economic crisis. These factors have made the availability of crude oil more unpredictable globally, leading market participants to anticipate a shortfall and pushing up oil prices. With the frequent occurrence of black swan events such as the China–US trade war in 2018 and the attack of a Saudi Arabian oil facility in 2019, the hedging effect of gold on crude oil was further enhanced. The COVID-19 pandemic commenced in early 2020, drastically harming the global economy and sharply reducing demand. In April 2020, oil prices declined by a historically large amount. However, as the pandemic was contained and the economy slowly recovered, oil prices started to increase in the middle of the year. After the outbreak of the Russian–Ukrainian conflict in 2022 and the Israel–Hamas conflict in 2023, Russia’s crude oil production was lower than before the conflict, geopolitical risks in the Middle East increased, and crude oil exports from neighboring countries such as Iran were limited, exacerbating global trade risks and crude oil market concerns about supply and demand, driving gold prices higher, but investor risk aversion gradually subsided as the situation eased. Figure 14 and Figure 15 reveal that the influence of gold weakened in 2016. In 2019, the out strength and in strength of gold reached its peak, and then fell back to a stable level. In 2023, it showed an upward trend and then declined. The above analysis shows that when the local political risk increases, the influence of gold as a hedging tool in the causal information network increases, and the investment hedging effect on the crude oil market is more obvious.

Compared with the previous study in reference [6], they found that the gold market dominates the other two variables in the intensity of causality, and it was found that the direction of causality of the variables changes during the COVID-19 pandemic. The result in this article supports the conclusion in reference [6] about the prominent position of gold in the network. In addition, we explore the nonlinear causal information intensity measured based on the transfer entropy method from the perspective of multiple time scales; it is concluded that the influence of gold on other variables in the short, medium, and long term and the influence of other variables are the largest, and the influence on other variables is particularly obvious in the medium term. During the COVID-19 pandemic, there were also significant fluctuations in the strength of its causal relationship, especially COMEX Gold. This result is a confirmation and supplement to previous studies.

### 3.3. Transmission Intermediary

**Figure 16:**
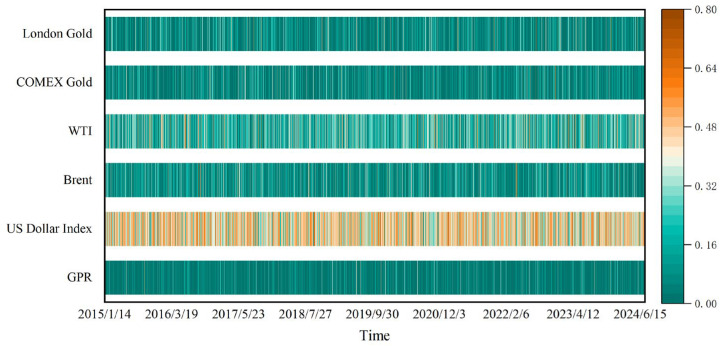
The betweenness centrality of each variable at the D1 scale varies with time.

**Figure 17:**
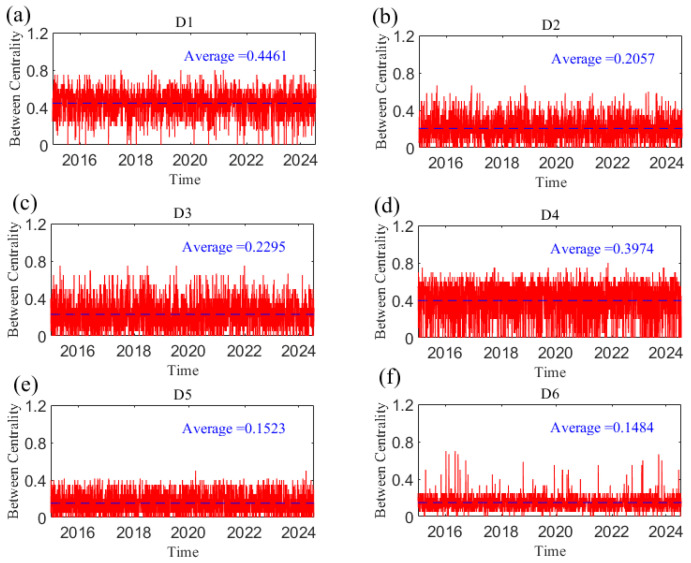
The betweenness centrality of US dollar index at the D1–D6 scales. (**a**) D1 scale; (**b**) D2 scale; (**c**) D3 scale; (**d**) D4 scale; (**e**) D5 scale; (**f**) D6 scale. Note: The dotted line in the figure represents the average value.

The results of the betweenness centrality of each variable at the D1 scale are shown in Figure 16. In general, the betweenness centrality of each variable is relatively stable over time. The US dollar has a significant mediating transmission effect compared with other variables, which provides a bridge function for the transmission of causal information among geopolitical risk and crude oil and gold prices. We now further investigate the dynamic characteristics of the mediating effect of the dollar in the causal information network at the short, medium, and long scales. Figure 17 shows the change in the betweenness centrality of the US dollar index over time at the D1–D6 scales. As the time scale increases, the mediating role of the dollar gradually weakens. It can be seen from the figure that in the Crimean conflict in 2014, the Chinese stock market crash in June 2015, the China–US trade friction in 2018, the outbreak of the COVID-19 epidemic in 2020, the Russia–Ukraine conflict in 2022, and the Israel–Hamas conflict in 2023, the dollar’s mediating ability is still greater at the D6 scale when it reaches a local peak, indicating that although the increase in time scale has weakened, the dollar still plays an important bridging role in the network when emergencies occur. This is because emergencies and uncertainties affect the international status of the dollar and, subsequently, affect the causal transmission of the entire system.

**Figure 18:**
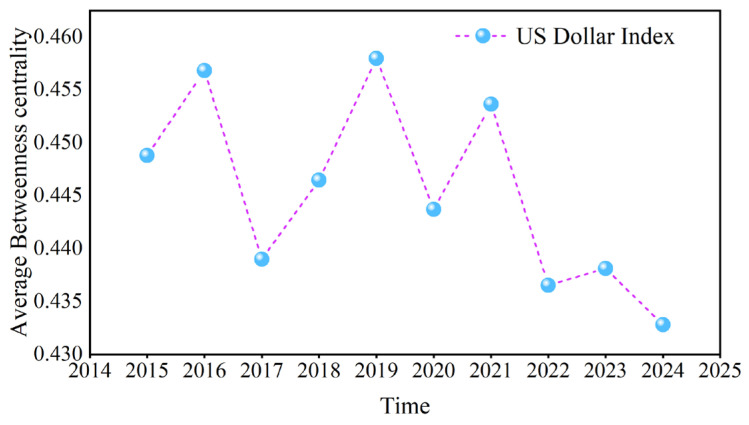
The annual average of the betweenness centrality.

Taking the betweenness centrality of the US dollar at D1 as an example, Figure 18 calculates the annual average to further analyze its change. The maximum value occurred in 2019, and the minimum value occurred in 2024. The China–US trade war in 2018 enhanced the ability of the US dollar to transmit information in the causal information network, whereas the COVID-19 pandemic, the Russian–Ukrainian conflict, and the Israel–Hamas conflict weakened this ability.

### 3.4. Complexity of Causal Information Networks

**Figure 19:**
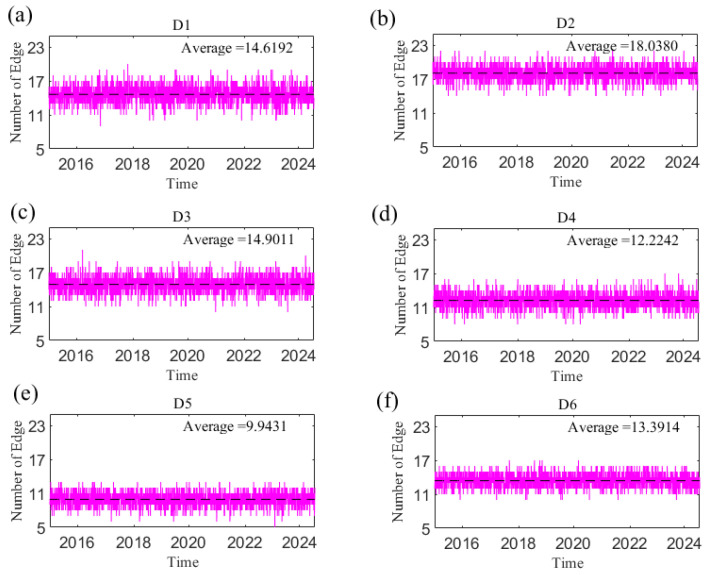
The number of the network edge changes with time at the D1–D6 scales. (**a**) D1 scale; (**b**) D2 scale; (**c**) D3 scale; (**d**) D4 scale; (**e**) D5 scale; (**f**) D6 scale. Note: The dotted line in the figure represents the average value.

In this paper, the overall complexity of the causal information network is reflected by the number of network edges. A greater number of edges indicates a stronger connection within the system. Figure 19 shows the change in the number of edges of the causal information network with time at the short, medium and long scales. The number of edges at each time scale is time-varying and decreases as the time scale increases; that is, the causal relationships among variables in the network weaken as the time scale increases.

The causal information network has the largest number of edges in the short term and the most complex overall structure, indicating that the overall linkage between geopolitical risks, US dollar, and crude oil and gold prices is stronger than that in the medium and long term. The spillover effects between geopolitical risks and crude oil and gold prices in reference [56] show that short-term returns and volatility spillover effects were stronger than long-term ones, and the overall spillover was mainly caused by short-term spillover effects. This is consistent with the results of this paper, indicating that the correlation among geopolitical risks and crude oil and gold markets based on causality and spillover relationships is significant in the short term.

### 3.5. The Information Transmission Speed of the Causal Network

**Figure 20:**
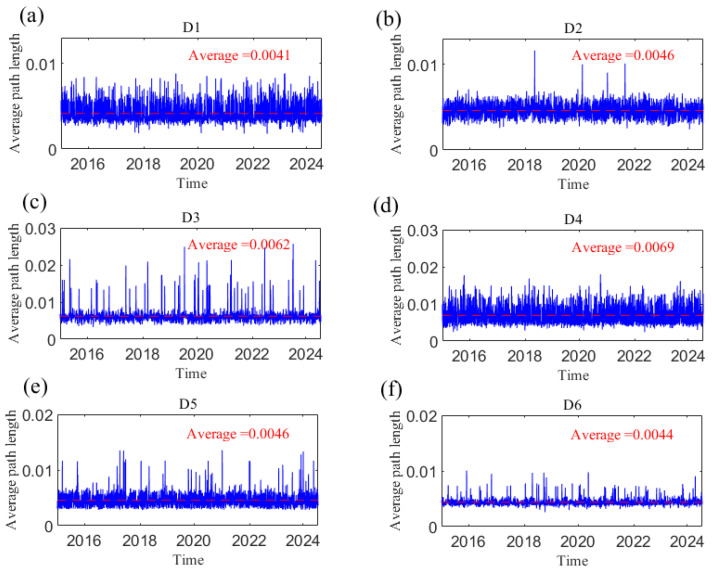
The average path length changes with time at the D1–D6 scales. (**a**) D1 scale; (**b**) D2 scale; (**c**) D3 scale; (**d**) D4 scale; (**e**) D5 scale; (**f**) D6 scale. Note: The dotted line in the figure represents the average value.

**Figure 21:**
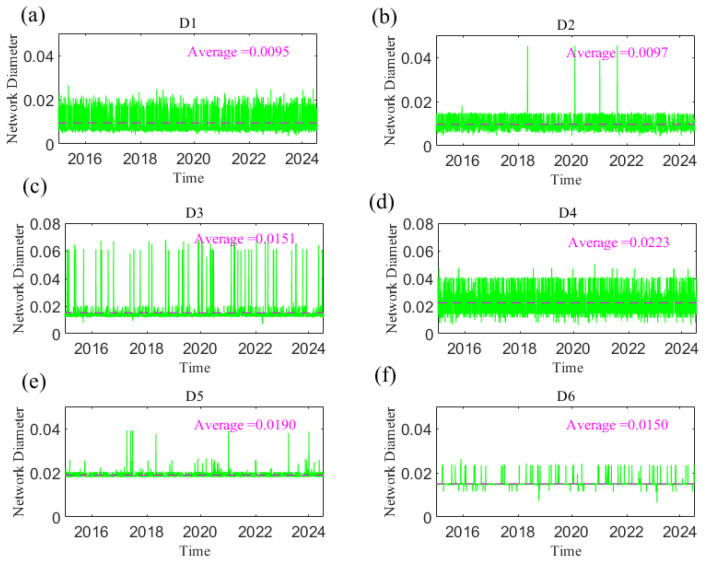
The network diameter changes with time at the D1–D6 scales. (**a**) D1 scale; (**b**) D2 scale; (**c**) D3 scale; (**d**) D4 scale; (**e**) D5 scale; (**f**) D6 scale. Note: The dotted line in the figure represents the average value.

The average path length (*L*) and network diameter (**d**) of the dynamic causal information network are measured; combined with typical financial events or extreme events, the dynamic characteristics of the causal information network structure are analyzed. Figure 20 and Figure 21, respectively, show the changes in *L* and *D* of the network with time at different time scales, reflecting the dynamic causal information transmission speed of geopolitical risk, the dollar index, and crude oil and gold prices at short, medium, and long scales. The smaller the two indices are, the greater the tightness of the network, indicating that the transmission speed of causal information is faster. The change trends of both parameters over the whole sample period were analyzed. For example, at scale D1, the *L* and *D* of the information transmission network are small, and the variations in both are relatively smooth, fluctuating at approximately 0.0041 and 0.0095, respectively. However, the index fluctuated sharply and reached its peak when uncertain events occurred, such as the Chinese stock market crash in June 2015, the China–US trade conflict in 2018, the outbreak of the COVID-19 pandemic in 2020, and the Russia–Ukraine conflict in 2022. Extreme events weaken the information transmission among variables. With increasing time scale, *L* and *D* at the short scale are smaller than those at the long scale, indicating that the impacts of geopolitical risk, the US dollar, and crude oil and gold prices are more rapid at the short scale. Obviously, the indices become increasingly stable as the time scale increases; that is, the causal relationship gradually stabilizes, and the integration of the network is improved. This finding shows that an increase in the time scale not only slows the transmission of causal information but also weakens the volatility of causality during emergencies and uncertainties among geopolitics, the US dollar, and crude oil and gold prices.

**Figure 22:**
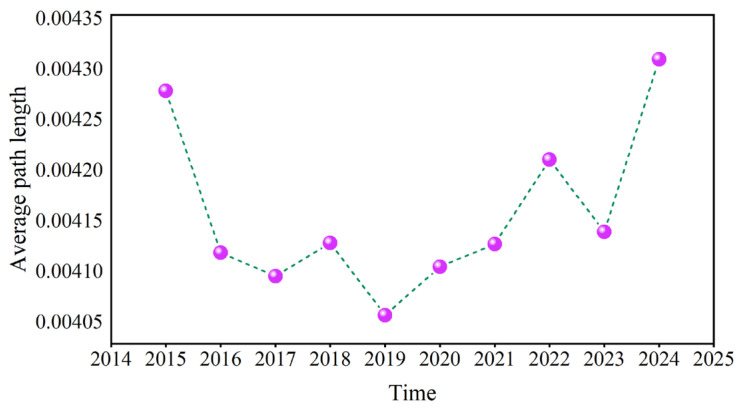
The annual average of the average path length.

**Figure 23:**
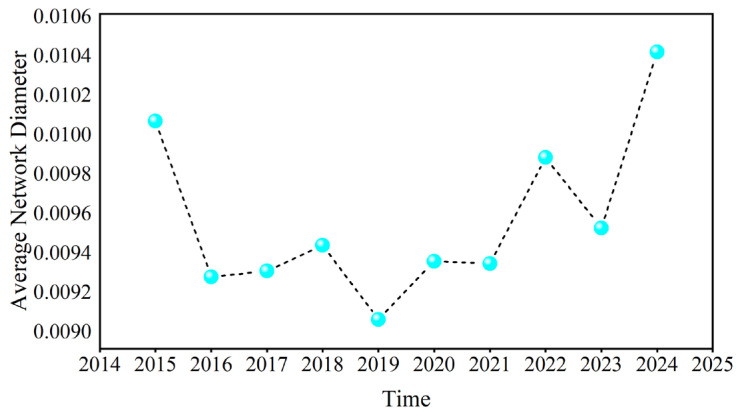
The annual average of the network diameter.

Figure 22 and Figure 23 present the annual averages of *L* and *D*. Overall, these values show a trend of first decreasing and then increasing, reaching the minimum value in 2019. These fluctuations were large during the COVID-19 pandemic, geopolitical conflicts, and when causal information transmission in the network became more rapid.

### 3.6. The Systematic Risk of the Causal Information Network

**Figure 24:**
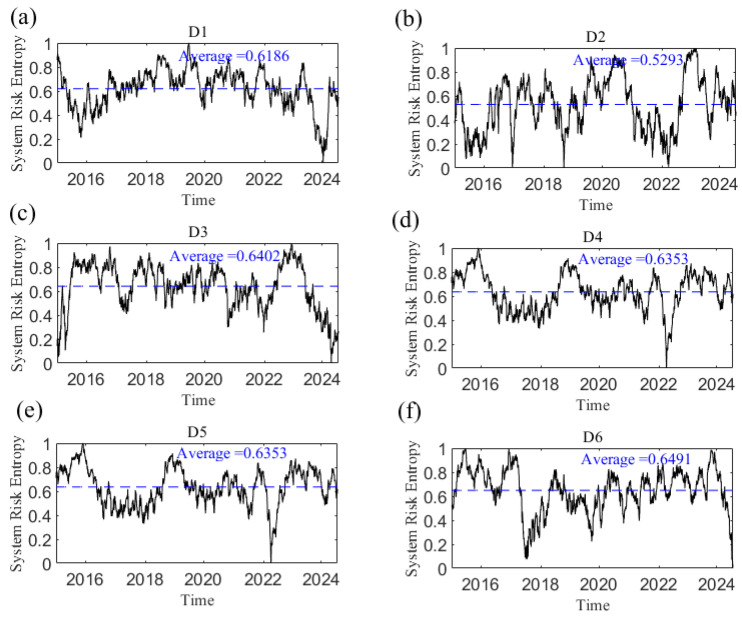
The dynamic system risk entropy at the D1–D6 scales. (**a**) D1 scale; (**b**) D2 scale; (**c**) D3 scale; (**d**) D4 scale; (**e**) D5 scale; (**f**) D6 scale. Note: The dotted line in the figure represents the average value.

Figure 24 shows the system risk entropy of the network at short, medium, and long scales. The larger the system risk entropy, the smaller the systematic risk. The network systematic risk is time-varying. Taking D1 as an example, the system risk entropy began to decline continuously from above the average value. After the Chinese stock market crisis in 2015 and the COVID-19 pandemic at the end of 2019, the local minimum value appeared, then rose gradually and fluctuated around the average level until the end of 2023 when the systematic risk reached the maximum, which was most likely due to the Israel–Hamas conflict. Additionally, from the overall trend of change, the short-scale (D1 and D2) network system risk entropy is the smallest, the medium-scale (D3 and D4) is higher, and the long-scale (D5 and D6) is the highest; that is, the average value of the short-scale network risk entropy is less than that of the long-scale network risk entropy. This finding indicates that systematic risk tends to decrease as the time scale increases.

**Figure 25:**
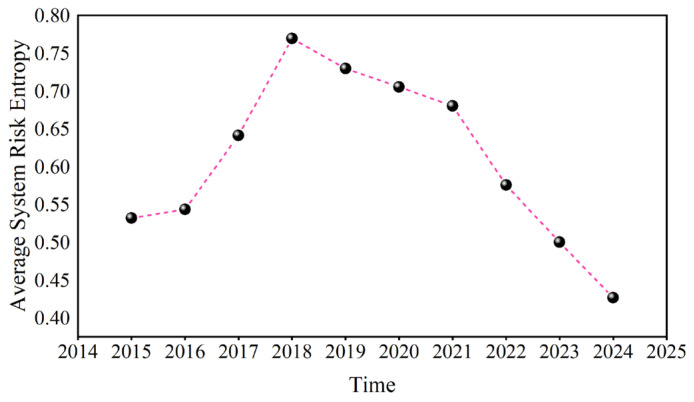
The annual average of the system risk entropy.

Figure 25 shows the annual average value of system risk entropy, which first tends to increase but then decreases. Since 2018, system risk entropy has continued to rise, indicating that systematic risk has been rising since the China–US trade war in 2018.

In the study of systematic risk, some researchers and scholars used the DY spillover index model [57] and CoVaR method [58,59], relying on data quality and parameter setting. If the data is non-stationary or there is extreme fluctuation, it affects the measurement effect of risk. This paper measures systematic risk based on entropy theory. System risk entropy can objectively measure the degree of confusion of the system, evaluate the uncertainty and complexity of different risk events, and has no requirements for parameter setting, which has a good effect on non-stationary data. And the results show that the systematic risk is the largest in the short term, which is consistent with the previous analysis of the overall complexity of the causal information network.

## 4. Conclusions and Discussion

We construct causal information networks that combine the MODWT model and the time-varying transfer entropy model with complex network theory to investigate the causality among GPR, the US dollar index, and crude oil and gold prices at multiple time scales. First, the original time series is decomposed into sub-time series at multiple scales via the MODWT model. Second, a sequence of transfer entropy matrices can be obtained according to the transfer entropy model and rolling window algorithm. Finally, a transfer entropy matrix can be transferred into a causal information network. The node is a sub-time series, and the transfer entropy value from one sub-time series to another is the weighted edge. The evolution of a single node and the dynamic structure of the network at multiple scales are analyzed. The conclusions and suggestions are as follows:

First, the variables that play important roles in the dynamic causal information network are identified from the perspective of different time scales. The influence of each variable in the causal information network has significant time scale dependence and evolves dynamically over time. Notably, gold has the strongest influence as a receiver and transmitter of causal information, especially at the medium scale, and different variables have different levels of importance at different time scales. This means that the interaction among geopolitics, the dollar, and bulk commodities is closely related to changes in time. The US dollar has the strongest mediating transmission ability in the causal information network and decreases with increasing time scale. The COVID-19 pandemic and geopolitical events have weakened this ability. Provide important early warnings for information changes.

Second, the structural characteristics of the causal information network system are analyzed. The results show that with as the time scale increases, the transmission speed of causality among variables becomes slower, and the network becomes more stable. At the short scale (D1), the transmission speed slowed during the Russian–Ukrainian conflict and the Israel–Hamas conflict. When an emergency occurs, the change trends of the average path and network diameter at different time scales can be found. For example, at the scale of 4–8 days, when the Chinese stock market crash and the outbreak of the COVID-19 pandemic occurred, they showed a significant rise. Systematic risk at the short scale is greater than systematic risk at the long scale; since the China–US trade war in 2018, systematic risk has continued to increase annually.

Third, when geopolitical risk is elevated, it often coincides with heightened activity in the gold market. The more obvious the hedging effect of gold as a hedging tool on the crude oil market, the stronger its dominant position within the causal information network.

The findings, analysis, and conclusions of this study offer significant insights and valuable references for relevant policy-making bodies and market investors.

First, this study suggests that investors should consider the time-varying characteristics of the causal relationships among geopolitical risk, the US dollar, and commodity markets, and the strength of influence among them at different scales. The adoption of a diversified investment strategy to reduce risks is recommended. Especially in times of uncertainty, more funds can be allocated to gold market because it contains greater profit opportunities and is a relatively safe investment option. Because the influence of emergencies is weakened and systematic risk is lower at the long scale, it is recommended that investors with conservative risk preferences choose long-scale investments in bulk commodity markets. Short-scale investors should focus more on market changes to adjust and improve their investment strategy in time.

Second, it is suggested that policy-makers establish a sound monitoring mechanism, strengthen the monitoring and analysis of changes in the strength of influence at different time scales, adjust policies accordingly to promote the stable development of the foreign exchange market and bulk commodity market, cut off risk infection, and ensure energy security. In particular, the US dollar, as a bridge of risk transmission between geopolitical risk and the crude oil and gold markets, must be focused on. In recent years, systematic risk has continued to increase, and policy-makers can reduce market uncertainty and volatility by encouraging long-scale investment to improve market stability or actively maintaining the stability of free trade and the international economic order to reduce the impact of external factors on the bulk market, thus decreasing market uncertainty and volatility.

Third, during periods of international political turbulence, the gold market presents more investment opportunities. Risk-oriented investors can increase their purchase of gold to gain income, thereby mitigating the impact of geopolitical risks on international crude oil supply and dealing with the uncertainty of financial market.

This paper studies only the causal information transmission between geopolitical risk, the US dollar index, the price of crude oil and the gold at multiple time scales through a wavelet transform model, and a transfer entropy causal network. Future research could explore the evolution of the linkage between geopolitical risk and other markets, such as bitcoin, financial currency, and mineral markets, at multiple scales. In addition, the advanced wavelet transforms model and advanced complex network theory, such as the high-order networks and multi-layer networks, could be used to investigate the multiscale risk transmission across markets in the future.

## Figures and Tables

**Table 1:** Distribution characteristics of each variable.

Series	Mean	Min.	Max.	Std. dev.	Kurt.	Skew.	J-B Test
GPR	116.279	9.490	540.830	52.863	11.859	2.011	0.001 ***
US dollar index	96.393	79.150	114.159	6.418	3.735	−0.488	0.001 ***
Brent crude oil future price	68.633	19.330	127.980	20.734	2.542	0.363	0.001 ***
WTI crude oil future price	64.015	−37.630	123.700	20.077	2.840	0.374	0.001 ***
COMEX gold price	1527.694	1051.900	2430.400	330.004	2.108	0.545	0.001 ***
London gold price	1527.656	1049.400	2427.300	329.727	2.104	0.543	0.001 ***

Note: *** denotes the 1% significance level. Min. means minimum values, Max. is maximum values, Std. dev. represents standard deviation values, Kurt. is kurtosis values, Skew. represents skewness values.

**Table 2:** Decomposition of time scale.

Time Scales	Time Frequency Domain	Scale
D1 D2	2–4 Days 4–8 Days	Long-scale
D3 D4	8–16 Days 16–32 Days	Medium-scale
D5 D6	32–64 Days 64–128 Days	Long-scale
S	More than 128 Days	Trend Level

**Figure A1:**
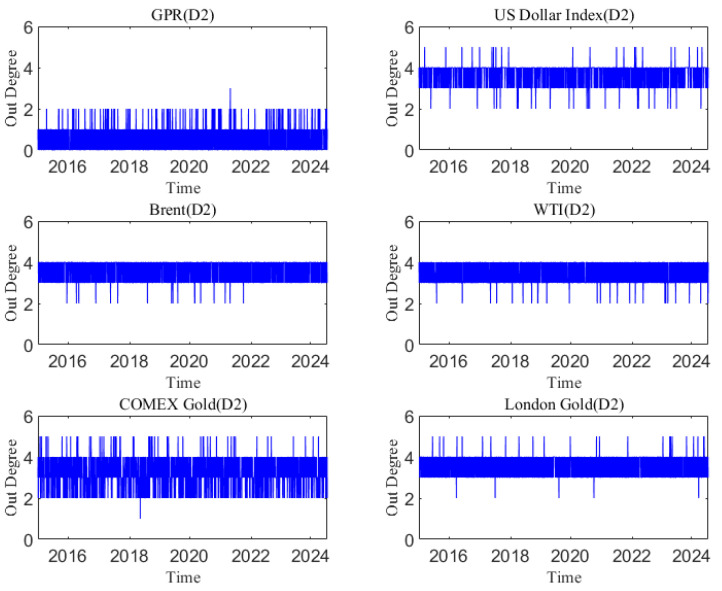
The out degree of each variable at the D2 scale varies with time.

**Figure A2:**
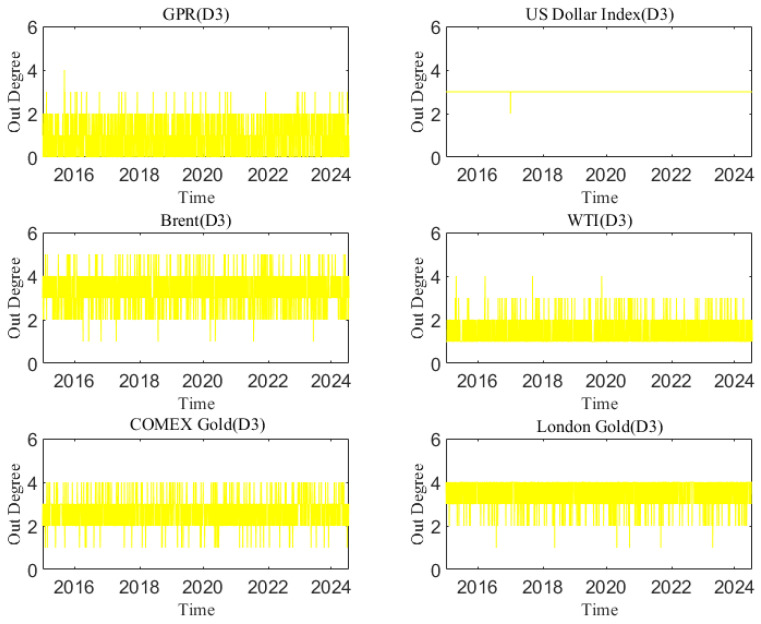
The out degree of each variable at the D3 scale varies with time.

**Figure A3:**
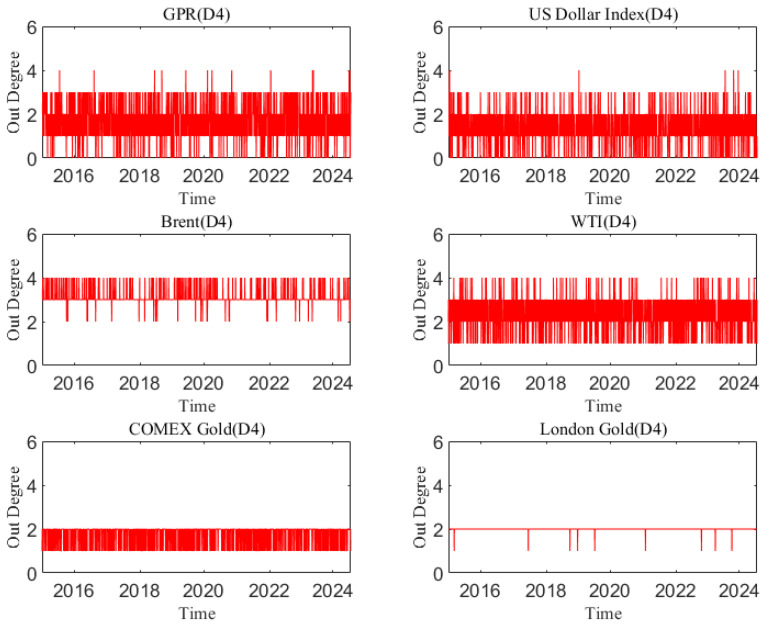
The out degree of each variable at the D4 scale varies with time.

**Figure A4:**
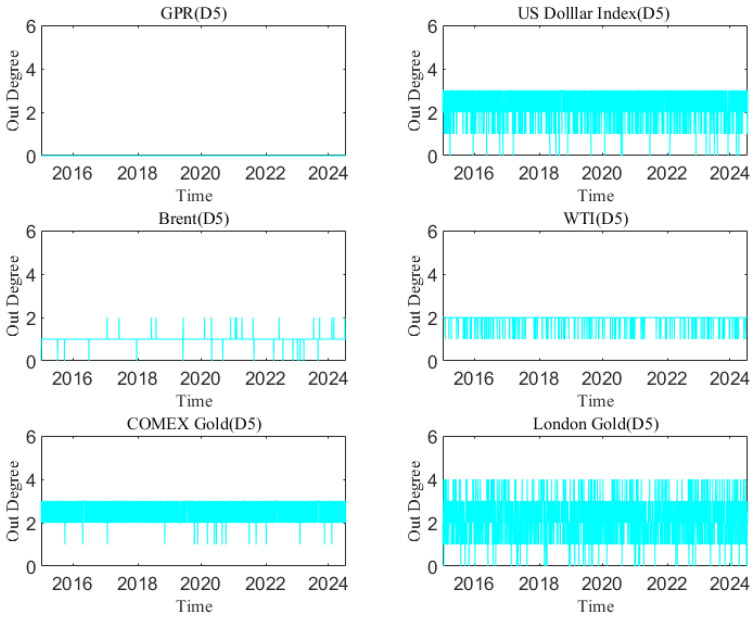
The out degree of each variable at the D5 scale varies with time.

**Figure A5:**
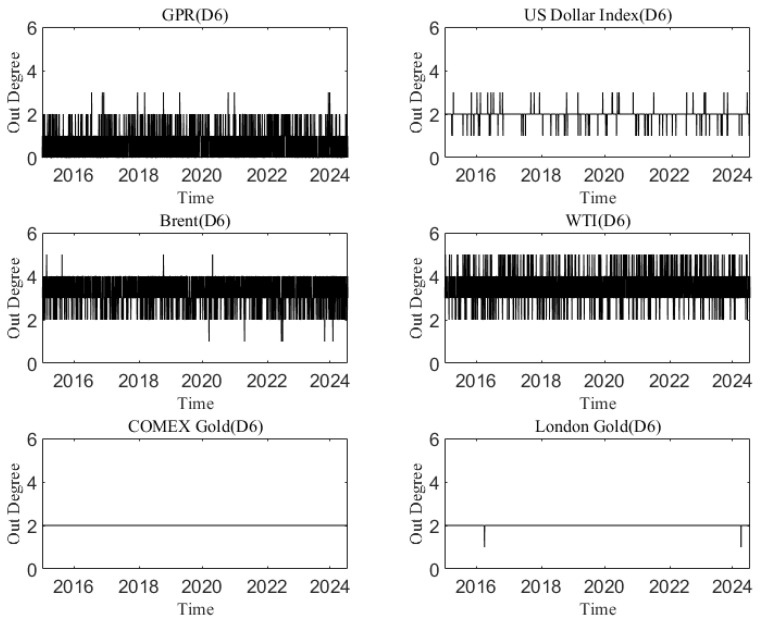
The out degree of each variable at the D6 scale varies with time.

**Figure A6:**
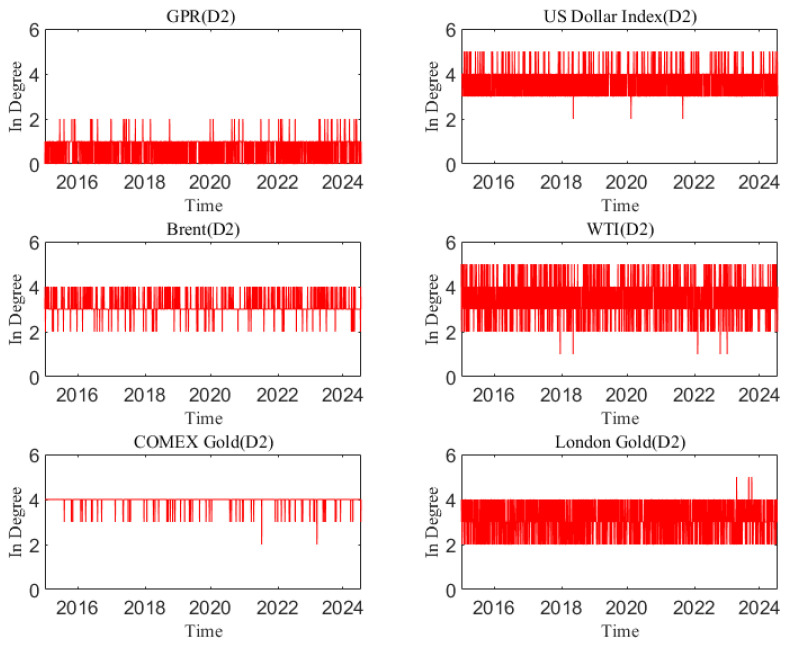
The in degree of each variable at the D2 scale varies with time.

**Figure A7:**
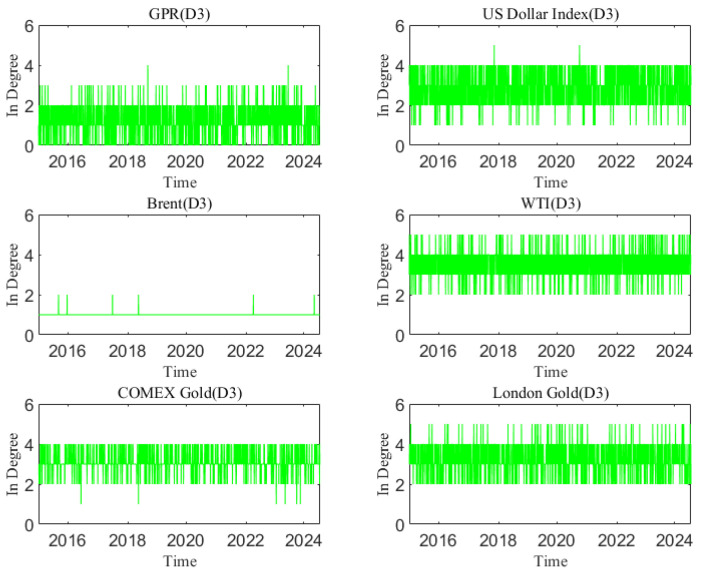
The in degree of each variable at the D3 scale varies with time.

**Figure A8:**
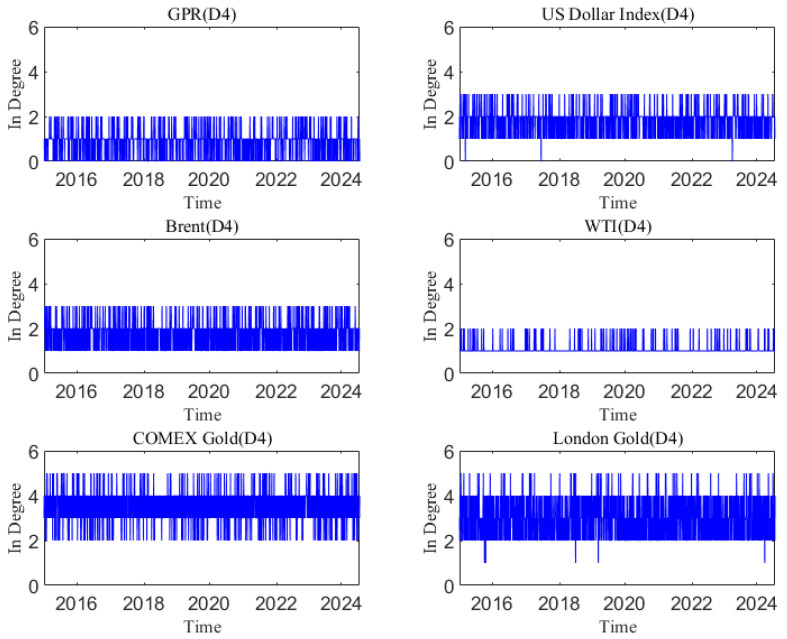
The in degree of each variable at the D4 scale varies with time.

**Figure A9:**
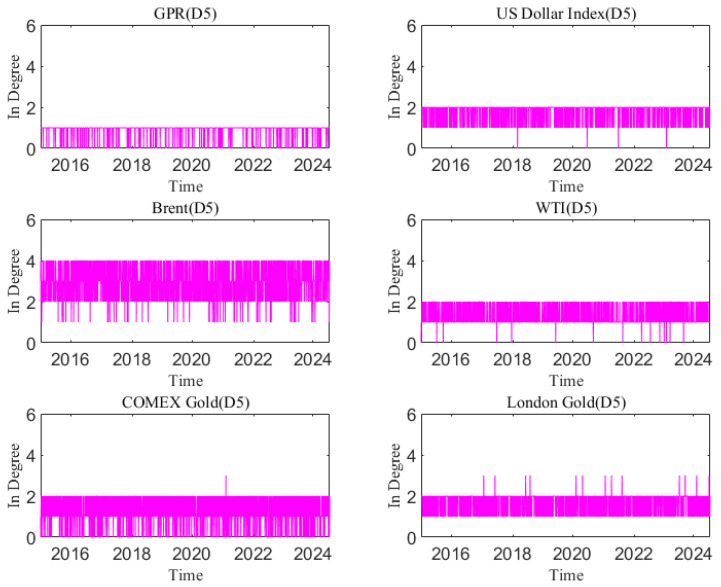
The in degree of each variable at the D5 scale varies with time.

**Figure A10:**
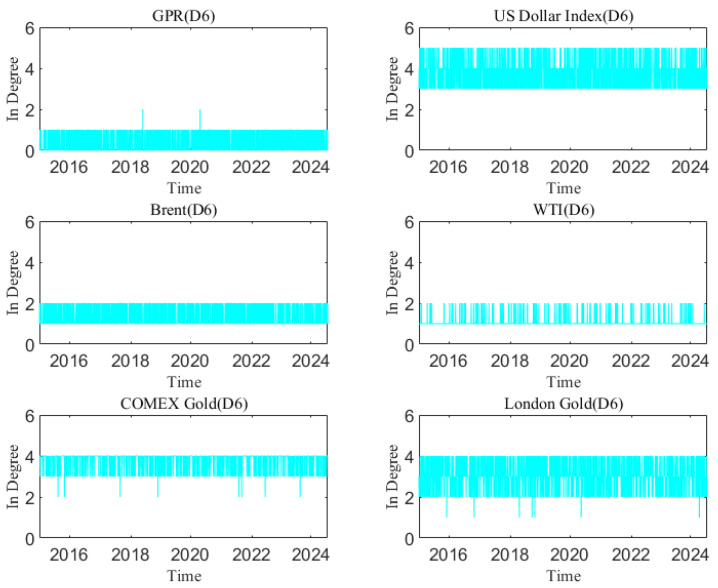
The in degree of each variable at the D6 scale varies with time.

**Figure A11:**
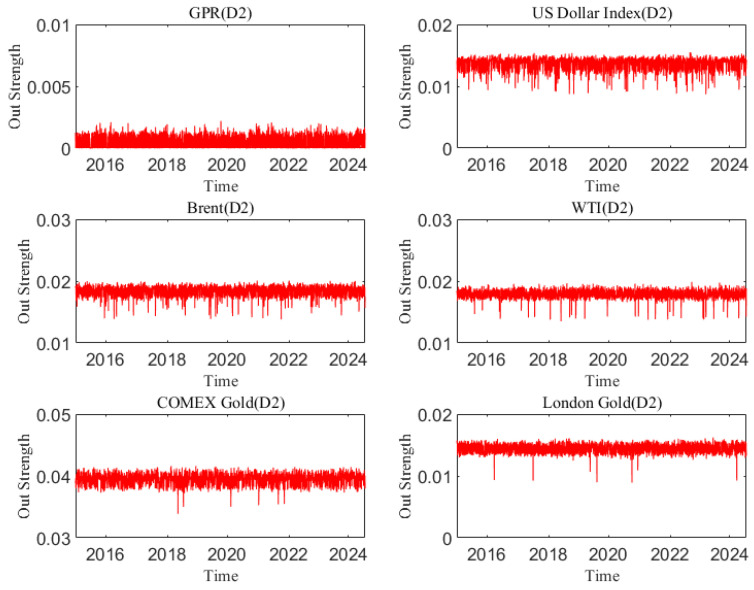
The out strength of each variable at the D2 scale varies with time.

**Figure A12:**
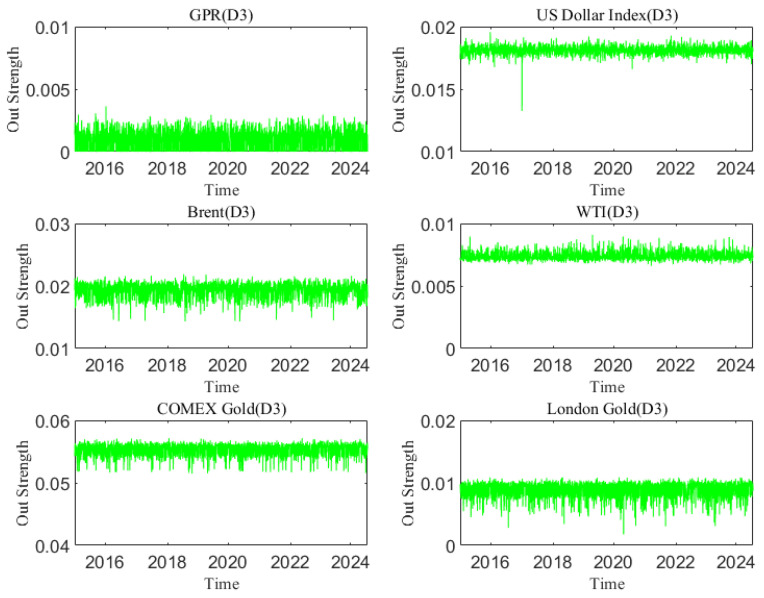
The out strength of each variable at the D3 scale varies with time.

**Figure A13:**
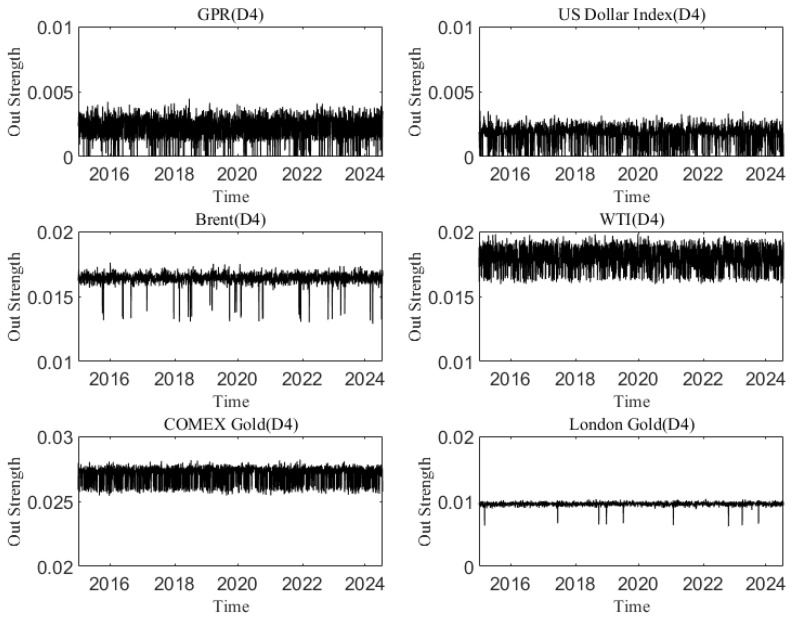
The out strength of each variable at the D4 scale varies with time.

**Figure A14:**
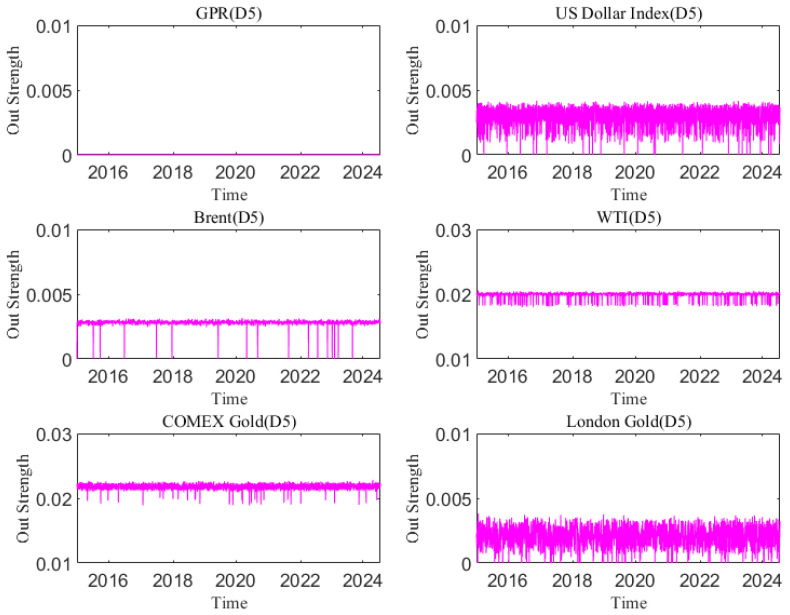
The out strength of each variable at the D5 scale varies with time.

**Figure A15:**
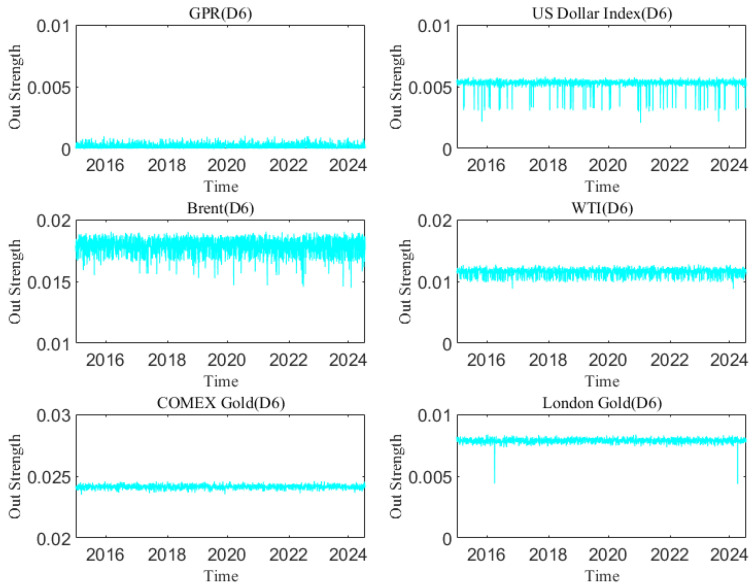
The out strength of each variable at the D6 scale varies with time.

**Figure A16:**
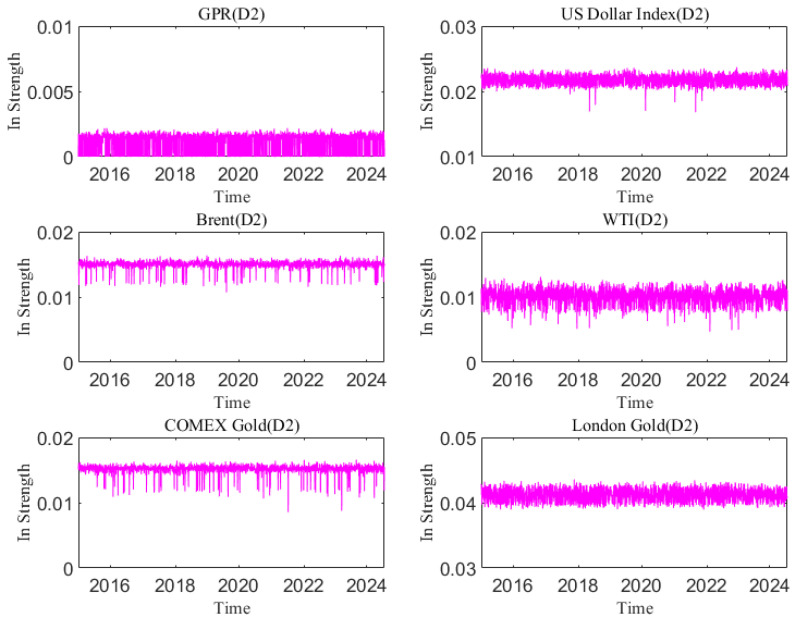
The in strength of each variable at the D2 scale varies with time.

**Figure A17:**
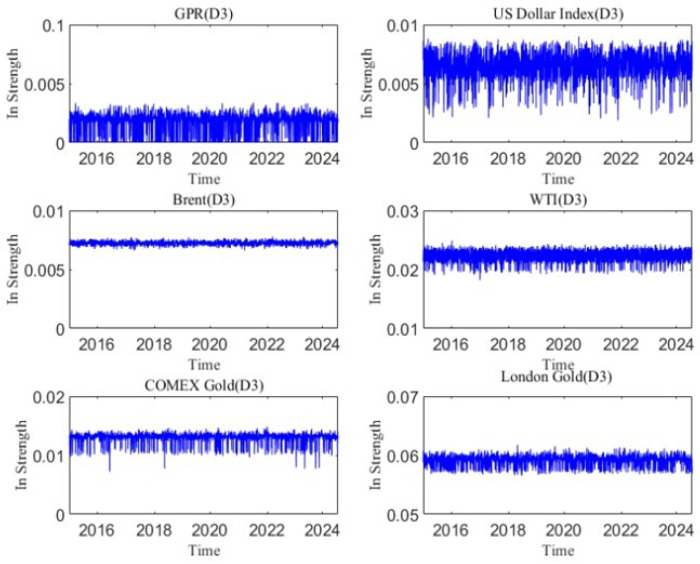
The in strength of each variable at the D3 scale varies with time.

**Figure A18:**
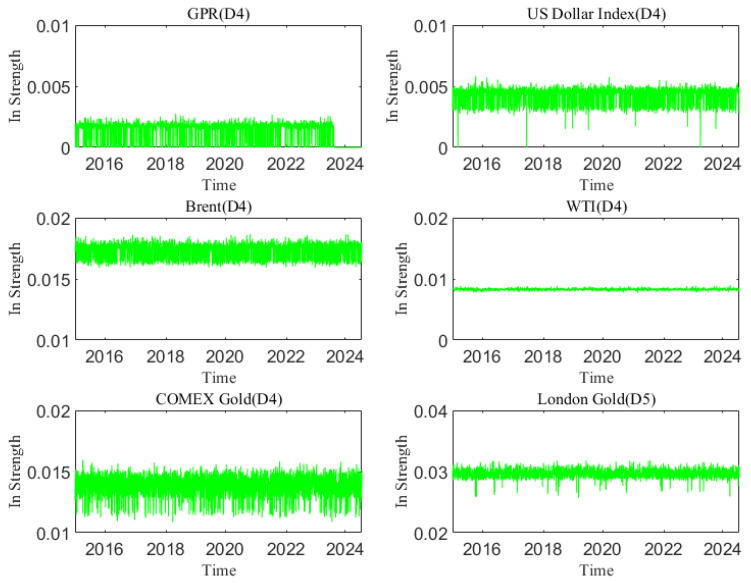
The in strength of each variable at the D4 scale varies with time.

**Figure A19:**
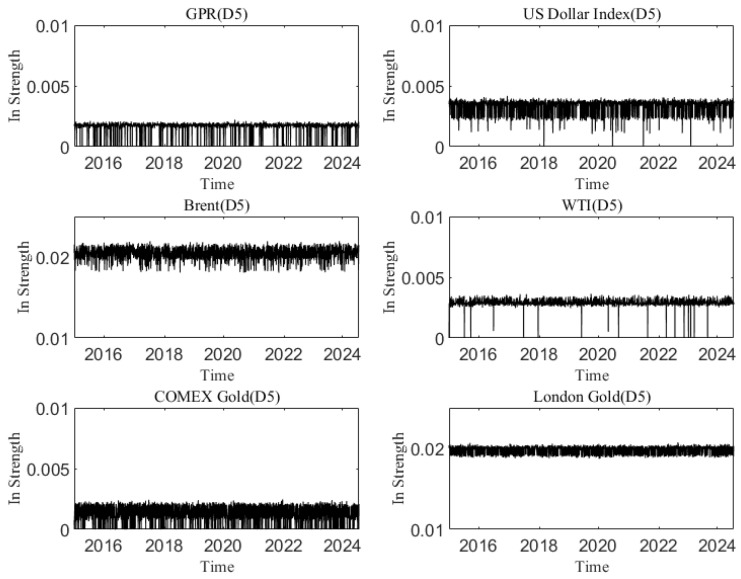
The in strength of each variable at the D5 scale varies with time.

**Figure A20:**
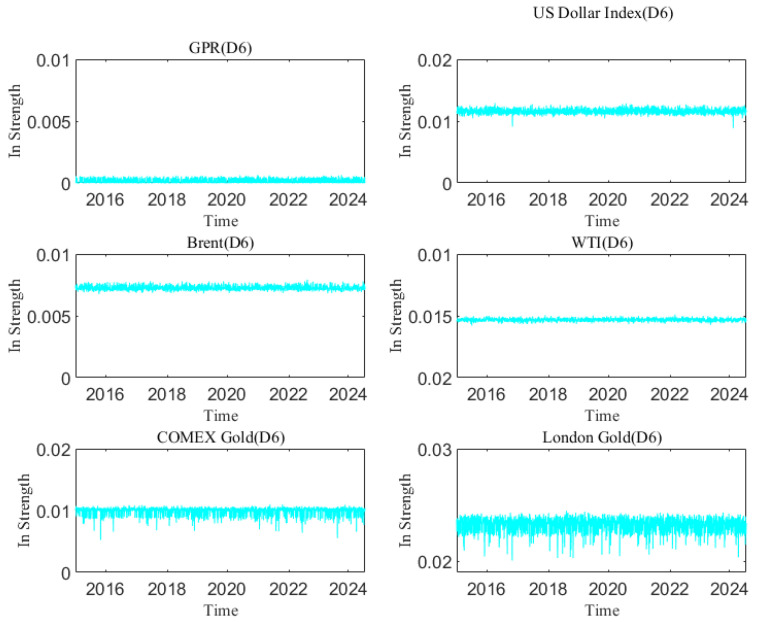
The in strength of each variable at the D6 scale varies with time.

## Data Availability

All the data were obtained from the website “https://www.matteoiacoviello.com/gpr.htm (accessed on 20 July 2024)” and the wind database.
